# The distribution and density of Huntingtin inclusions across the Huntington disease neocortex: regional correlations with *Huntingtin* repeat expansion independent of pathologic grade

**DOI:** 10.1186/s40478-022-01364-1

**Published:** 2022-04-19

**Authors:** Richard A. Hickman, Phyllis L. Faust, Karen Marder, Ai Yamamoto, Jean-Paul Vonsattel

**Affiliations:** 1 Department of Pathology, Memorial Sloan Kettering Cancer Center, 1275 York Avenue, New York, NY 10065 USA; 2 Department of Pathology & Cell Biology, Columbia University Irving Medical Center, New York Presbyterian Hospital, 630 W 168th Street, New York, NY 10032 USA; 3 Department of Neurology, Columbia University Irving Medical Center, New York, USA; 4 Taub Institute for Research On Alzheimer’s Disease and the Aging Brain, Columbia University Medical Center, 710 West 168th Street, New York, NY 10032 USA

**Keywords:** Huntington disease, HD, Huntingtin, Inclusion, Aggregation, Cortex, Striatum, Trinucleotide repeat, Neuropathologic staging

## Abstract

Huntington disease is characterized by progressive neurodegeneration, especially of the striatum, and the presence of polyglutamine huntingtin (HTT) inclusions. Although HTT inclusions are most abundant in the neocortex, their neocortical distribution and density in relation to the extent of CAG repeat expansion in the *HTT* gene and striatal pathologic grade have yet to be formally established. We immunohistochemically studied 65 brains with a pathologic diagnosis of Huntington disease to investigate the cortical distributions and densities of HTT inclusions within the calcarine (BA17), precuneus (BA7), motor (BA4) and prefrontal (BA9) cortices; in 39 of these brains, a p62 immunostain was used for comparison. HTT inclusions predominate in the infragranular cortical layers (layers V-VI) and layer III, however, the densities of HTT inclusions across the human cerebral cortex are not uniform but are instead regionally contingent. The density of HTT and p62 inclusions (intranuclear and extranuclear) in layers V-VI increases caudally to rostrally (BA17 < BA7 < BA4 < BA9) with the median burden of HTT inclusions being 38-fold greater in the prefrontal cortex (BA9) than in the calcarine cortex (BA17). Conversely, intranuclear HTT inclusions prevail in the calcarine cortex irrespective of *HTT* CAG length. Neocortical HTT inclusion density correlates with CAG repeat expansion, but not with the neuropathologic grade of striatal degeneration (Vonsattel grade) or with the duration of clinical disease since motor onset. Extrapolation of these findings suggest that HTT inclusions are at a regionally-contingent, CAG-dependent, density during the advanced stages of HD. The distribution and density of HTT inclusions in HD therefore does not provide a measure of pathologic disease stage but rather infers the degree of pathogenic *HTT* expansion.

## Introduction

Hallmarks of many neurodegenerative diseases are the relative selective vulnerability of specific cell types to injury and the accretion of pathologic protein inclusions in the central nervous system (CNS). In Huntington disease (HD), the cardinal pathologic features are the worsening, topographic, degeneration of the neostriatum (caudate nucleus, putamen, and nucleus accumbens), in which the medium spiny neuron is the most vulnerable cell type, and the appearance of huntingtin inclusions due to abnormal polyglutamine (polyQ) expansion of the huntingtin protein (HTT) [13, 63, 64]. Although the brunt of neurodegeneration in HD is concentrated in the neostriatum, morphometric studies of extra-striatal brain regions, such as the frontal neocortex, white matter, thalamus and brainstem, collectively emphasize widespread degeneration in the HD brain [10, 22, 23, 38, 49–52]. Yet despite the well-described neurodegenerative changes that occur in the HD brain, data on the neuroanatomic distributions of pathologic inclusions are lacking. Furthermore, how these proteins relate to striatal neurodegeneration remain uncertain [49–52]. If aggregated HTT and its oligomers are toxic, then the distribution of these inclusions may be clinically significant and their removal would be therapeutically relevant [2, 3, 13, 16, 18, 41, 59, 66].

Wild-type huntingtin has a myriad of functions throughout life (reviewed in [54]) and is expressed in neurons of the central nervous system, predominating in the cytoplasm with a small proportion also present within the nucleus [14, 17, 60]. Antibodies directed against the *N*-terminal region of HTT have revealed visible pathologic HTT aggregates (inclusions) within the HD brain and in murine model systems of *HTT* expansion [13, 18, 25, 31, 37, 63]. In the HD brain, these inclusions may be found within neuronal nuclei (neuronal intranuclear inclusions) and outside the nucleus (extranuclear inclusions) as seen within the perikaryal cytoplasm or the cytoplasm of axons and dendritic spines as dystrophic neurites. Human postmortem studies have shown that HTT inclusions are most abundant in the neocortex, prevailing in layers III, V and VI, which correspond to the same layers that are most vulnerable to neuronal loss [13, 21, 58]. Brains with juvenile-onset HD show a greater number of intranuclear inclusions, whereas brains with adult-onset HD have mostly extranuclear inclusions [3, 9, 13, 18, 42]. But beyond this, immunohistochemical studies that have investigated the distribution of HTT aggregation in the cortex vary considerably, likely due to small sample sizes and variation of antibodies utilized. Herndon et al. applied the 1C2 antibody, which targets the polyQ stretch of the HTT protein, to a series of 19 HD brains and found widespread inclusions in the brains without noticeable differences of abundance between neocortices [25]. Conversely, Van Roon-Mom et al. utilized the EM48 antibody and found a higher density of inclusions in the prefrontal cortex (BA9) than in the posterior frontal cortex (BA4/6) in 7 HD brains [61]. The EM48 antibody was raised against a.a. 1–256 of the HTT protein with the epitope mapping to the C-terminus of HTT exon 1 [65]. Becher et al. found step-wise increases in intranuclear HTT inclusion density with higher CAG repeat expansions in the cortex of 20 HD brains using a variety of HTT-specific antibodies and demonstrated intercortical variability of intranuclear inclusion density [1]. Finally, Gutekunst et al. found that EM48-labeled HTT inclusions are present in the cortex before onset of clinical signs and are larger in grade 4 (n = 2) than in grade 1 brains (n = 2) [18]. Cortical differences in HTT densities were noted within individual HD brains but a defined distribution in 12 HD brains was not established.

Attempts to address any differential neurodegeneration in the HD neocortex have provided mixed results. Three morphometric studies that examined the macroscopic thickness of the cortex of coronal sections of the cerebral hemispheres demonstrated diffuse, even, and symmetric cortical thinning, except for relative sparing of the medial temporal lobe by Halliday et al. [10, 20, 61]. In contrast, one of the first volumetric studies on human HD tissue claimed that the occipital neocortex is the most severely afflicted in terms of neuronal loss and that the pattern of progression within the neocortex may be caudal to rostral [48]. Caudo-rostral progression of cortical thinning has also been proposed by Rosas et al. in addition to hemispherical asymmetry, with the left cerebral hemisphere being more involved than the right by structural MRI [47, 48]. However, the small series of stereological studies in HD by Rüb and others show similar degrees of neuronal loss (32–33% loss compared with control brains) between the calcarine cortex and other cortical regions arguing against differential neuronal loss in the neocortex [24, 51].

The entire neocortex sends corticostriatal glutamatergic projections to the neostriatum in a topographic manner, a subset of which synapse onto the medium striatal spiny neurons [32, 33]. Given these topographic projections to the neostriatum and the progression of caudal to rostral, medial to lateral, and dorsal to ventral degeneration in HD, if HTT is neurotoxic to cortical neurons, one might predict lower abundances of inclusions in the frontal neocortices that project to the dorsomedial head of the caudate nucleus and the sensorimotor regions of the putamen and a greater burden in the occipital and parietal neocortices that project to the more caudal aspects of the caudate nucleus in early stage HD brains [8, 19, 32, 57]. This assumption would also be dependent on the premise of uniform neuronal loss across the cortex. Alternatively, if neuronal degeneration is predominant within caudal neocortices, such as the calcarine cortex, then the density of HTT inclusions may be least in the occipital cortex in contrast with more rostral cortices at postmortem provided there is clearance of HTT following neuronal death.

With this in mind, we sought to characterize the neocortical distribution of pathologic inclusions in 65 HD brains. These were selected to encompass the spectrum of severity in HD, including juvenile- and adult-onset individuals with CAG repeat expansions ranging from 38- 71 repeats and ages at death between 24 and 89 years-of-age. HTT inclusions and their relationship to CAG repeat expansion, metrics of disease burden (Vonsattel grade, CAG age product (CAP) score, length of time from motor onset to death) were investigated. The selected distribution of HTT inclusions in the brain, correlation with CAG repeat expansion and relationship to grade and symptom duration are discussed.

## Materials and methods

### Ethical approval

All brain donors were consented for autopsy and brain donation. All procedures performed were in accordance with the ethical standards of the institution and with the 1964 Helsinki declaration and its later amendments.

### Study design

The 65 brains of this study had a pathologic diagnosis of HD and known *HTT* CAG repeat size. All brains were processed using the standardized protocol in place at the New York Brain Bank as outlined previously [28, 62]. The half brain or whole brain that was assigned for neuropathologic diagnosis, was fixed by immersion in 10% neutral-buffered formalin solution for approximately two weeks and then cut and blocked as per the standardized New York Brain Bank protocol as previously described [62]. Both cerebral hemispheres were sliced in the coronal plane at 0.3 cm intervals. The Brodmann map was utilized for blocking cortical regions to allow for consistent comparisons of neocortical regions across multiple brains [7]. Formalin-fixed tissue blocks of BA17 (calcarine), BA7 (precuneus), BA4 (motor cortex/ leg region), and BA9 (prefrontal cortex/ superior frontal gyrus) were then processed, embedded in paraffin wax, sectioned perpendicular to the cortical surface at 7 μm thickness and collected on positively-charged slides. These four neocortical areas were chosen because they are aligned parasagittally enabling for caudal to rostral assessment without significant lateral deviation and are away from watershed territories so as to be less influenced by peri-mortem hypotension.

### Immunohistochemical staining

Paraffin sections were immunostained on an automated immunostaining platform using a DAB with or without alkaline phosphatase dual staining system on a Roche Ventana staining platform (Table 1). Hematoxylin counterstains were performed. We used the 2B4 antibody which targets amino acids 1–82 of the N-terminal end of HTT to study HTT inclusions; this antibody has been validated in human HD brains previously [11, 12, 25, 28]. To corroborate the distribution of HTT, we also examined sections stained with an antibody directed against p62 in a subset of cases (n = 39). P62 is an autophagy adaptor protein that binds to ubiquitinated HTT for subsequent degradation by macroautophagy [4, 35].

**Table 1:** Details regarding primary immunohistochemical stains used in this study

Antibody	Company	Catalogue number	Dilution	Primary antibody incubation time (minutes)	Protocol and dilution	Platform	Chromagen
Huntingtin/ p62 double stain	Millipore/ Abcam	MAB5492/ ab207305	1:2000 each	32/24	64 min (CC1)	Roche Ventana Instrument	Brown- huntingtin Red- p62
P62	Abcam	Ab207305	1:2000	24	64 min (CC1)	Roche Ventana Instrument	Brown- p62

### Assessment of inclusion density

Immunohistochemically stained slides were blinded by demographics, *HTT* CAG repeat length, clinical disease duration, and neuropathologic grade before examination and analysis. All slides were reviewed by one board-certified neuropathologist (RAH). HTT and p62 inclusions were defined as a protein aggregate visible by light microscopy that were immunolabeled by their respective antibody (anti-HTT or anti-p62). Intranuclear inclusions were defined as inclusions localized to within the hematoxylin-counterstained nucleus; extranuclear inclusions were defined as any inclusion outside of the nucleus, i.e. within the visible perikaryal cytoplasm of a cell or as inclusions that appear within the neuropil. Since HTT inclusions predominate in the infragranular layers, quantification of inclusions was performed in cortical layers V–VI. For HTT quantification, inclusions labeled with brown chromogen with the huntingtin/ p62 double stain were analyzed. For p62 quantification, inclusions labeled with brown chromogen with the p62 single stain were analyzed. For both HTT and p62 quantification, three photomicrographs per cortical region (BA17, BA7, BA4, BA9) of layers V–VI were obtained at an original magnification of 200x. Then, using ImageJ 1.52P and the Cell counter plugin, intranuclear and extranuclear inclusions were manually counted per image field (RAH) [56]. The total inclusion count of three representative fields was calculated and then divided by the total field area to determine the inclusion density (number of inclusions per mm^2^).

### Metrics of disease burden

Several measures have been proposed to assess disease severity. One reliable and widely used measure of neurodegeneration within the neostriatum is the Vonsattel grading system; all brains studied were diagnosed and graded either by JPV or RAH [64]. Another measure of disease burden is the CAP score (age × (CAG repeat length–33.66)/432.3326) [40, 67]. Age-at-death provides a measure of the length and severity of exposure to mutant *HTT* at the end of life. For individuals with a known age-of-onset of HD motor signs (n = 22), the duration of clinical disease was calculated by subtracting the age of clinical onset of motor signs from the age at death.

### Statistical analysis

Statistical analyses were performed on GraphPad Prism version 9.1.2. Normality tests were performed on all numerical datasets using the D’Agostino Pearson test. For data that was not normally distributed, non-parametric tests were utilized. Average values are presented as means ± standard deviation (SD) if normally distributed, or as median ± interquartile range (IQR) if non-normal. Multiple comparisons of median inclusion densities between brain region were initially assessed by pairwise comparison using the Freidman test, followed by post-hoc Dunn’s multiple comparison test for regional differences. Correlational analyses between CAG repeat length and inclusion density were performed using Spearman’s rank correlational coefficient. Regression analyses assume linearity and significant differences in slopes of regression lines were assessed between BA9 and BA17 using Prism’s built-in function that is equivalent to the ‘analysis of covariance’ (ANCOVA) analysis [1]. A *P* value < 0.05 was deemed significant (*) and a *P* value < 0.01 was considered highly significant (**); *P* values < 0.001 and < 0.0001 were also designated on graphs as *** and ****, respectively.

## Results

### Cohort

The mean age at death of the 65 brain donors was 56.3 years ± 14.1 years (SD) and 31 (47.7%) individuals were men. The Vonsattel grade of HD neuropathologic changes ranged from 1–4 with a mean grade of 3 ± 0.83 (SD). The median CAG repeat expansion was 45 (IQR: 4).

### Inclusion patterns and intracellular localization

**Fig. 1:**
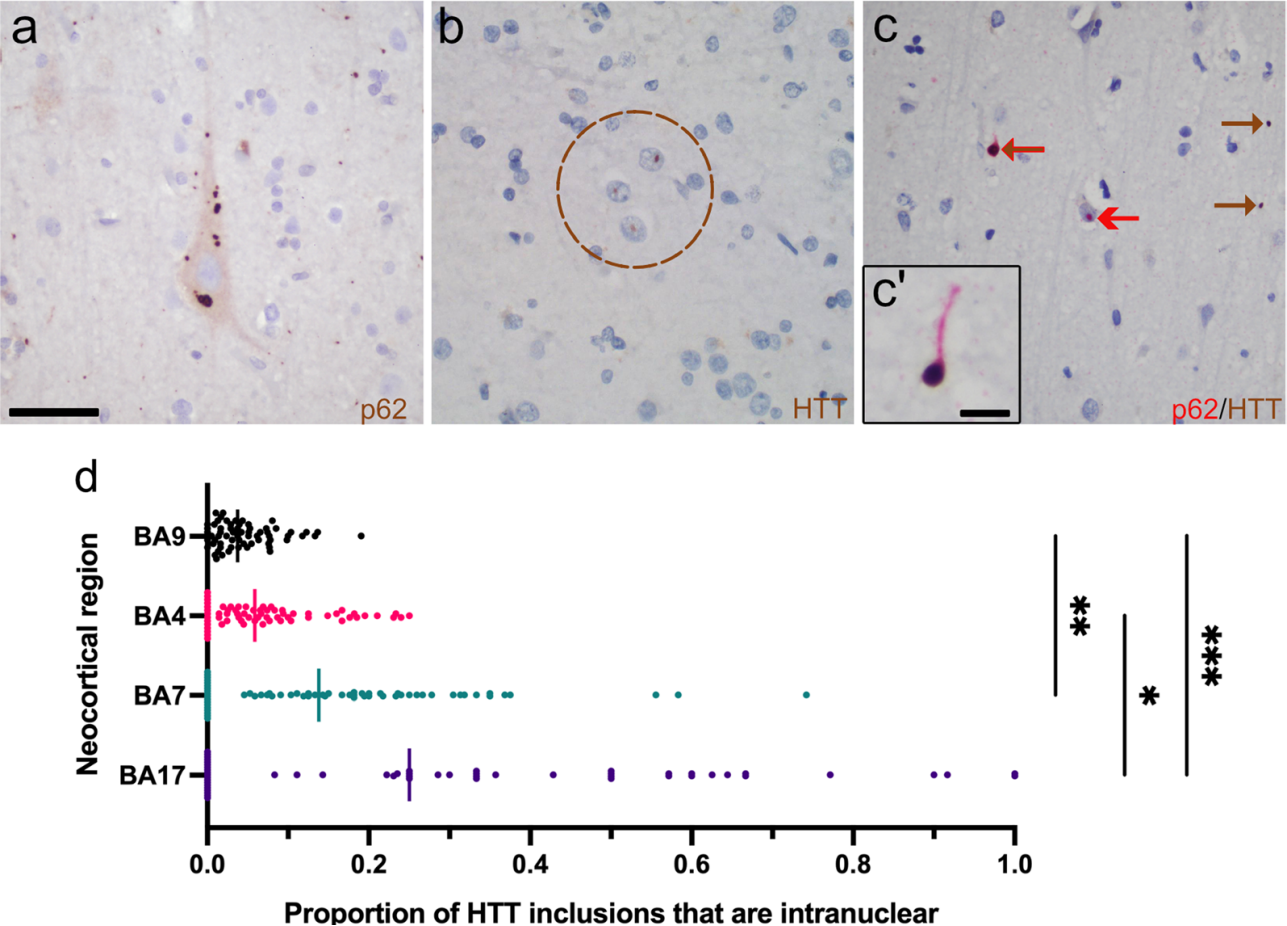
Intracellular distribution of HTT and p62 inclusions within HD. **a** Numerous HTT or p62 cytoplasmic inclusions can be found within some neurons, such as in this Betz cell of a 48-year-old woman with 45 CAG repeats. **b** Intranuclear HTT inclusions are occasionally found in nuclear clusters, particularly in individuals with > 50 CAG repeats. This example was from the calcarine cortex (BA17) of a 30-year-old man with 61 CAG repeats. **c** Dual staining for HTT (brown)/p62 (red) highlights p62 colocalization with HTT and wrapping of p62 around HTT. Inset (**c′**) shows a fortuitous dystrophic neurite with a long extension of p62 that emanates from and enwraps an extranuclear HTT inclusion. **d** The proportion of intranuclear HTT inclusions relative to total HTT inclusions varies in the neocortex and prevails in caudal neocortices, especially the calcarine cortex (BA17). Vertical lines indicate the median value for the respective neocortical region. Note that in the instance of BA17 where the absolute number of inclusions is least of all four cortical regions, only 54 individuals could have calculated ratios because 11 brains had no inclusions in BA17. Brown arrows indicate HTT labeling; red arrows indicate p62 labeling. Brown arrows with a red edge indicate p62 colocalizing with HTT. Scale bars: **a**–**c**: 50 µm, **c′**: 10 µm

As previously described, both intranuclear and extranuclear HTT and p62 inclusions predominated within neocortical layers V-VI and III, with some inclusions present in the immediate subcortical white matter [13, 18]. In the adult-onset HD cases, extranuclear inclusions vastly outnumbered the intranuclear inclusions overall (Fig. 1a, b). In instances where the perikaryal cytoplasm could be discerned around its respective nucleus, inclusions were either found within the cytoplasm or within the nucleus, but never concomitantly in both compartments. Intranuclear inclusions were nearly always singular, whereas perikaryal inclusions could be singular or multiple (Fig. 1a). Occasionally, groups of closely spaced cortical neurons contained intranuclear HTT or p62 inclusions (Fig. 1b). Using dual immunoperoxidase labeling against HTT and p62, a subset of HTT inclusions were enwrapped by p62, a protein that is important in autophagic removal of aggregated proteins (Fig. 1c).

The ratio of intranuclear inclusions/total inclusions increases in more caudal cortices (*P* < 0.0001, Kruskall Wallis test; BA17 vs. BA9, *P* = 0.007; BA7 vs. BA9, *P* = 0.013, post-hoc Dunn test, Fig. 1d). The median ratio of intranuclear inclusions/ total of the calcarine cortex was 0.25 ± 0.52 (IQR), whereas that of the prefrontal cortex was 0.038 ± 0.057 (IQR). Thirteen of fifty-four individuals had predominantly intranuclear HTT inclusions (i.e., a ratio > 0.5) in the calcarine cortex, and only one of these individuals had juvenile-onset HD. Therefore, although intranuclear HTT inclusions are generally considered less prominent than extranuclear HTT inclusions in adult-onset HD brains, there are regional differences in the localization of HTT inclusions in the HD cortex [13, 18].

### Neocortical variation in HTT and p62 inclusions

**Fig. 2:**
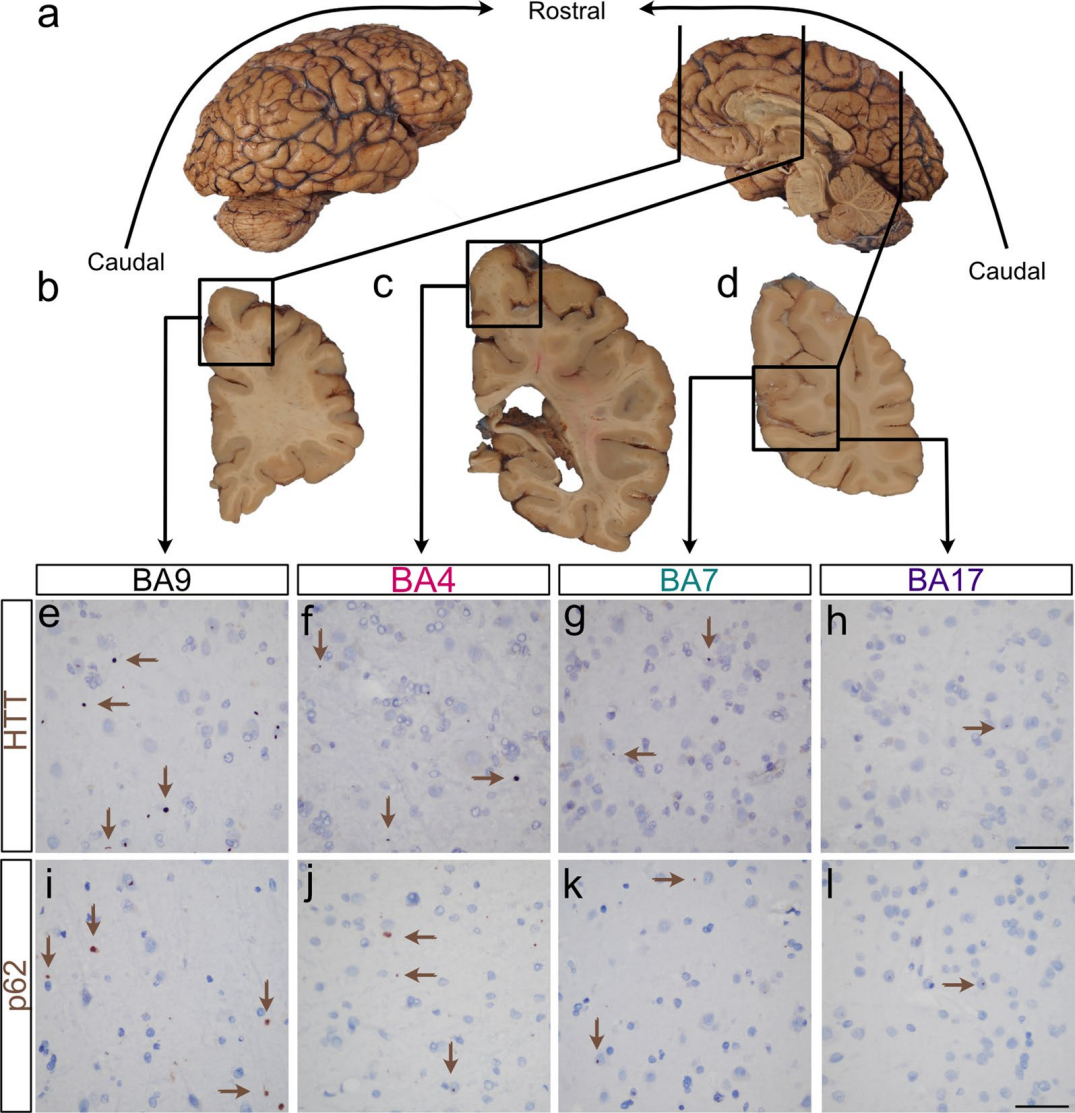
HTT and p62 inclusion density follows an increasing caudal to rostral gradient in parasagittal neocortical regions of HD. **a** Macroscopic photographs of the formalin fixed right half brain of a 56-year-old man with adult-onset HD (CAG: 47/21) demonstrating the lateral and medial aspects. The brain (fresh weight: 981.6 g) appeared diffusely small. Vertical lines indicate the plane of section to obtain the coronal slices of images (**b**)–(**d**). **b–d** Select coronal slices of this brain from rostral to caudal with rectangles showing the Brodmann areas of interest, BA9 (superior frontal cortex, **b**), BA4 (motor cortex, **c**) and BA7/ BA17 (precuneal cortex and calcarine/visual cortex, respectively, **d**). **e**–**l** Immunohistochemical stains against HTT (**e**–**h**) and against p62 (**i**–**l**) demonstrating gradually increasing inclusion densities from BA17, BA7, BA4 to BA9. Brown arrows indicate a subset of the inclusions seen in these micrographs. Original magnifications: **e**–**l**: 400×, scale bars of **e–l**: 50 µm

**Fig. 3:**
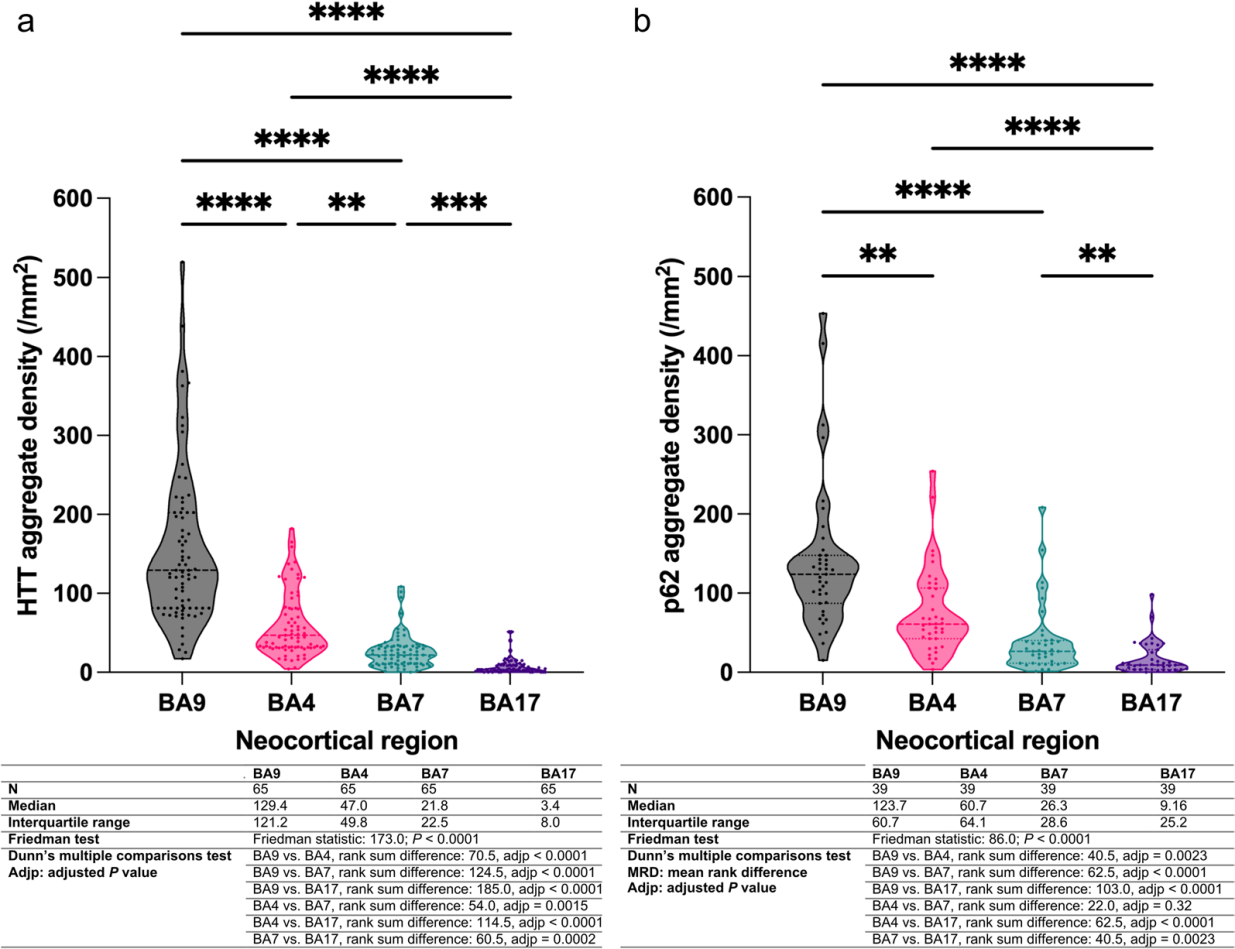
Violin plots of total HTT inclusion densities (**a**) and p62 inclusion densities (**b**) across neocortical regions showing successive increases from caudal to rostral cortices (BA17 → BA9). Statistical tables for these respective proteins are provided beneath each graph. **a**, **b** Dots indicate individual cortical regions; horizontal dashed lines indicate median values while horizontal dotted lines indicate the first and third quartile values

We next quantified total HTT inclusion density (i.e. both intra- and extranuclear HTT inclusions) in the infragranular layers (layers V/VI) of neocortical areas BA17, BA7, BA4, and BA9 of the 65 brains. We found that the median density of HTT inclusions was least in the calcarine cortex (BA17) and that this density sequentially increased in a caudal to rostral direction with a nearly 38-fold difference in median inclusion density between BA17 and BA9 (Figs. 2e-h, 3a). Similarly, an increasing caudal to rostral gradient was found with total p62 inclusion density; there was a 14-fold difference in median p62 density between BA17 and BA9 (Figs. 2i–L, 3b, N = 39). Significant correlations between the HTT and p62 inclusion densities were found in BA17, BA7 and BA9 (r = 0.54, *P* = 0.0004, r = 0.49, *P* = 0.0017, r = 0.40, *P* = 0.012, respectively, n = 39).

### Neocortical HTT and p62 inclusion densities correlate with CAG expansion

**Fig. 4:**
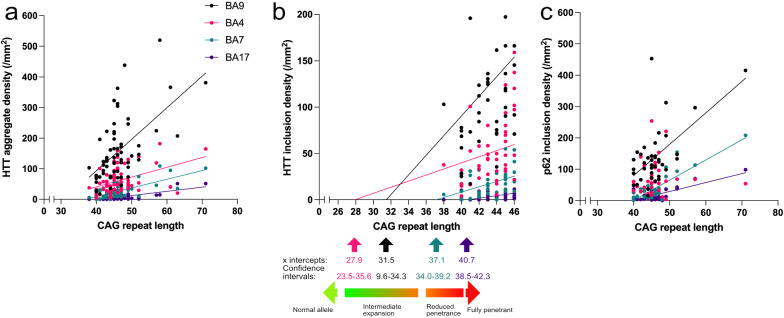
Bivariate regression plots showing correlations between CAG repeat expansion and neocortical HTT/ p62 inclusion density. **a** HTT inclusion density correlates with CAG repeat expansion and increasing gradient of HTT burden along a caudal to rostral direction. **b** The x-intercepts of the extrapolated linear regression lines between HTT and CAG imply that inclusions may be formed in individuals with intermediate CAG expansion. **c** p62 inclusion density significantly correlates with CAG repeat expansion in BA17, BA7 and BA9

Total HTT inclusion density correlated with CAG repeat length of the 65 patients in all four brain regions studied (BA17: r = 0.49, *P* < 0.0001; BA7: r = 0.60, *P* < 0.0001; BA4: r = 0.44, *P* = 0.0003; BA9: r = 0.53, *P* < 0.0001, n = 65, Spearman’s rank correlation coefficient, Fig. 4a). The increase in HTT inclusion density per CAG repeat rose in a caudal to rostral direction (BA9 vs. BA17, F = 23.1, *P* < 0.0001, see methods for statistical analysis). Extrapolation of the regression lines demonstrated an incremental decrease in the x-intercept of the regression lines from caudal to rostral cortices (BA17, BA7, BA4, BA9, Fig. 4b). Although the confidence intervals are broad and assuming linearity in the regression lines, the extrapolation raises the possibility that HTT inclusions might be present in the prefrontal cortex in individuals with CAG expansions between CAG_22-35_ and in the precuneus at CAG_34-39_ with the x-intercepts of these cortices corresponding to the intermediate expansion and reduced penetrance ranges, respectively.

We next examined the relationship between p62 and CAG repeat expansion in a subset of cases (n = 39). Significant correlations were present between p62 inclusion density and CAG repeat expansion in BA17, BA7, and BA9 but not with BA4 (BA17: r = 0.36, *P* = 0.026, BA7: r = 0.39, *P* = 0.014; BA4: r = 0.23, *P* = 0.16; BA9: r = 0.40, *P* = 0.011, n = 39; Spearman’s rank correlation coefficient, Fig. 4c). Similar to the relationship between HTT density and CAG repeat expansion, the slopes of the p62 lines of BA17, BA7, and BA9 were progressively steeper from caudal to rostral (BA9 vs. BA17, F = 9.9, *P* = 0.0024, see methods for statistical analysis).

### HTT neocortical inclusion density accords with CAG repeat expansion regardless of disease grade

The Vonsattel grading system is a five-point neuropathologic scale used to measure the extent of degeneration within the striatum from symptomatic HD patients [64]. HD brains without any evidence of striatal degeneration, when assessed with conventional clinical neuropathologic methods, are assigned 0/4, while those with severe neuronal loss and widespread gliosis in the striatum are designated 4/4. Therefore, if the striatum degenerates from grades 0 to 4 over the clinical disease course, this system could also potentially assess whether neocortical HTT inclusion density changes with disease progression.

**Fig. 5:**
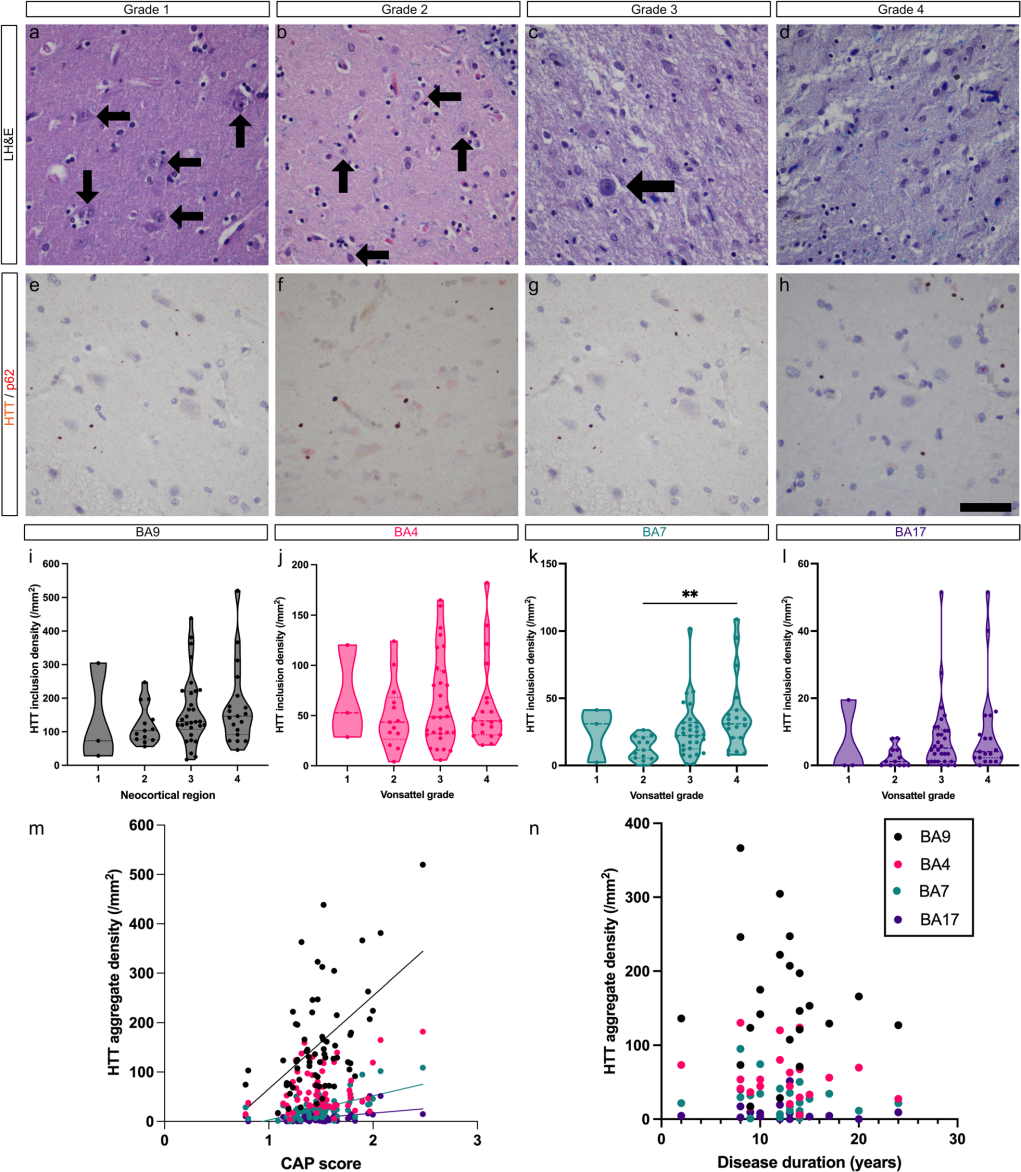
**a**–**d**: Selected examples of brains that ranged from grade 1 to grade 4. **a**–**d** High power image of the dorsal head of the caudate nucleus. Arrows indicate neurons and demonstrate increasing neuronal loss with higher grades and worsening gliosis in higher grades. **e**–**h** HTT/p62 immunostains demonstrate similar inclusion densities between the prefrontal neocortices (BA9) despite increasing grade. **i**–**l** Violin plots showing that HTT inclusion density is stable across neocortical regions, except between grades 2 and 4 in BA7. Horizontal dashed lines indicate the median. **m**–**n** Bivariate regression plots demonstrate correlations between CAP score and HTT inclusion density in BA17, BA7 and BA9 (m, n = 65), however, no correlations were found between the clinical disease duration and HTT inclusion densities (n) across BA17, BA7, BA4 and BA9 (n = 22). Scale bar: 50 µm

There were no significant differences in neocortical HTT inclusion density across grades 1–4 in cortical regions assessed (Fig. 5e-l, n = 65), except for the precuneal cortex (BA7) between grades 2 and 4 (*P* = 0.0028, n = 65, Dunn’s multiple comparison test, Fig. 5k). However, there were no differences of HTT inclusion density between grades 1 and 4, which would be counterintuitive if HTT inclusions accumulate with progression of disease.

Significant correlations were found between the CAP score and total HTT inclusion densities in BA9, BA7, and BA17 (BA9: r = 0.42, *P* = 0.0006, BA4: r = 0.19, *P* = 0.14, BA7: r = 0.47, *P* < 0.0001, BA17: r = 0.43, *P* = 0.0003, n = 65, Spearman’s rank correlation, Fig. 5m). However, no correlation was found between clinical disease duration, i.e. time from motor onset to death, and HTT inclusion density in any of these four cortices (Fig. 5n, n = 22). Overall, these findings raise the possibility that there is no significant accumulation of HTT inclusions with disease progression but rather that the inclusion density is at a steady state and driven by the extent of CAG repeat expansion. Consistent with this, correlations between inclusion density and CAG expansion size were repeatedly stronger than with CAP score across all 65 individuals with known CAG repeat lengths (BA17: r = 0.50 vs. 0.43; BA7: r = 0.62 vs. r = 0.47; n = 65, BA4: r = 0.46 vs. r = 0.19; BA9: r = 0.48 vs. r = 0.42, n = 65).

## Discussion

By utilizing 65 well-characterized HD brains, we found a wide variation of the distribution and density of HTT inclusions across the human HD neocortex. Importantly, HTT inclusion density follows an increasing caudal to rostral gradient (BA17 < BA7 < BA4 < BA9) that is irrespective of cortical cytoarchitecture (heterotypical vs. homotypical). HTT inclusion density correlates with CAG repeat expansion and rostral cortices have greater increases in inclusion density per CAG repeat than caudal cortices. Although intranuclear inclusions are overall more frequent in the juvenile HD cortex with higher CAG repeat expansions than in adult-onset HD, we found that the ratio of intranuclear HTT inclusion density / total HTT inclusion density increases in more caudal cortices in HD brains regardless of the size of CAG repeat expansion and can predominate in the calcarine cortex in adult-onset HD brains [13, 18]. Therefore, the subcellular localization of HTT inclusions in the cortex is regionally dependent. Furthermore, there is an apparent mutual exclusion of the cellular localization of the inclusions, which are either within the nucleus or cytoplasm.

Pathogenic trinucleotide repeat expansion of the *HTT* gene increases the length of the polyglutamine tract of the HTT protein and results in aberrant splicing of the *HTT* transcript in a CAG repeat length dependent manner [53]. This produces the small polyadenylated *HTT1a* transcript that encodes the highly aggregation-prone and pathogenic exon 1 HTT protein and may initiate aggregation within certain compartments of the cell, depending on its subcellular concentration [45, 46]. In no instance were inclusions found concomitantly in both the nucleus or cytoplasm of a neuron. This mutual exclusion may be because inclusion formation in one subcellular compartment may recruit exon 1 HTT from another compartment [36]. In this model, the formation of intranuclear inclusions would depend on whether exon 1 HTT could cross the nuclear membrane, which would be contingent on its size and governed by the length of the polyQ tract. Genome wide association studies have indicated that somatic CAG repeat expansion drives the onset and progression of HD and somatic CAG expansion would result in increased levels of exon 1 HTT with longer polyQ tracts [39, 43]. The relatively higher density of intranuclear inclusions compared with extranuclear inclusions in BA17 than other regions of the cortex, may therefore reflect differences in somatic CAG repeat expansion between these cortical regions.

The burden of HTT inclusions predominate in the infragranular layers of the cortex (layers V/VI), which was our rationale to focus our study on these layers [13, 18]. Studies have demonstrated a relationship between the size of the CAG repeat expansion and the extent of HTT aggregation [34, 55]. Scherzinger demonstrated that polyglutamine expansion above 30 was sufficient to cause HTT aggregation in vitro and that increasing size of polyglutamine stretches and exon 1 HTT concentration, increased aggregation [55]. An increased propensity for self-propagation by mutant HTT with increased polyQ lengths partly explains the correlation between CAG length and HTT inclusion density. However, the cause for regional differences in inclusion density is not clear. One possible cause of the increasing caudal to rostral gradient of HTT burden is that there is a gradient of somatic CAG repeat expansion across the neocortex that influences HTT inclusion density, consistent with the concept of variable CAG repeat expansions across the cortex [44]. Another possibility is that increased neuronal loss of more caudal cortices earlier in the disease would result in fewer neurons at the end of life and reduced HTT burden relative to rostral cortices at the time of autopsy provided there is clearance of HTT inclusions during life [47, 48].

Pathologic grading of HD is widely used to assess the severity of neurodegeneration for an individual postmortem. The general finding that the HTT inclusion burden does not differ across grade (except for one instance in BA7) suggests that there is a steady burden during the clinical disease. Ubiquitinated and HTT inclusions are indeed present in prodromal HD gene carriers, as well as in non-HD individuals with pathogenic *HTT* expansion and amyotrophic lateral sclerosis (ALS) [12, 15, 18, 26, 27]. Collectively, this evidence implies that the presence and caudal to rostral distribution of pathologic HTT inclusions are a phenotypic expression of *HTT* gene CAG expansion that does not necessarily signify clinical disease or pathologic grade. This contrasts with other more common neurodegenerative diseases (e.g., primary age-related tauopathy, Alzheimer disease, Parkinson disease), where pathologic inclusions have a tendency to accumulate over time and involve successive brain regions allowing for reliable disease staging [5, 6, 29].

There are several limitations to this study. Firstly, there was limited clinical annotation to many of these brains, partly because HD is rare and the brain bank relies upon referrals from across the nation to acquire this large collection often with limited clinical information. Secondly, only one HTT immunostain was employed in this study and may not have bound all HTT species. However, the p62 immunostain confirmed the presence of an increasing caudal to rostral gradient of inclusions and essentially supported the data gained from the HTT immunostains. Thirdly, like other autopsy studies, this work is correlative and cross-sectional thus preventing the intravitam study of HTT production and clearance, i.e., turnover, within individuals.

## Conclusions

In summary, we have demonstrated a wide variation in HTT inclusion density within the human HD neocortex and a variable propensity for aggregation for different neocortices. The uncertainty remains as to whether these inclusions are toxic to the CNS. A higher burden of HTT inclusions in the neocortex did not unequivocally associate with higher grades of disease. Instead, these data suggest that the presence and distribution of HTT inclusions is a gain-of-function phenotypic expression of *HTT* gene expansion. The spectrum of pathologic effects of pathogenic *HTT* gene expansion has now expanded to include not simply the HD neurodegenerative phenotype, but also to include abnormalities during neurodevelopment, including a greater propensity for developmental malformations in the brain, as well as ALS [12, 27, 28, 30]. Further investigation as to the biological mechanism for why some cortical regions have disparate burdens of HTT inclusions and whether this signifies variable resistance to HTT accumulation across cortices may reveal possible therapeutic avenues for patients with pathogenic *HTT* gene expansion.

## Data Availability

Data is available upon reasonable request.

## Abbreviations

ALS: Amyotrophic lateral sclerosis
ANCOVA: Analysis of covariance
BA: Brodmann area
BA4: Brodmann area 4 (frontal lobe, paracentral)
BA7: Brodmann area 7 (parietal lobe, precuneus)
BA9: Brodmann area 9 (frontal lobe, prefrontal cortex)
BA17: Brodmann area 17 (occipital lobe, primary visual cortex)
CAP: CAG age product
CNS: Central nervous system
HD: Huntington disease
HTT: Huntingtin
IQR: Interquartile range
polyQ: Polyglutamine
SD: Standard deviation
